# MRI of High-Glucose Metabolism Tumors: a Study in Cells and Mice with 2-DG-Modified Superparamagnetic Iron Oxide Nanoparticles

**DOI:** 10.1007/s11307-015-0874-0

**Published:** 2015-07-07

**Authors:** Xiu Hong Shan, Peng Wang, Fei Xiong, Ning Gu, Hui Hu, Wei Qian, Hao Yue Lu, Yu Fan

**Affiliations:** Department of Radiology, The Affiliated Renmin Hospital of Jiangsu University, Zhenjiang, 212002 China; Oncology Institute, The Affiliated Renmin Hospital of Jiangsu University, Zhenjiang, 212002 China; State Key Laboratory of Bioelectronics, Jiangsu Key Laboratory of Biomaterials and devices, School of Biological Science and Medical Engineering, Southeast University, Nanjing, 210009 China; Medical college, Jiangsu University, Zhenjiang, 212013 China

**Keywords:** 2-Deoxy-d-glucose, Superparamagnetic iron oxide, Glucose metabolism, Breast cancer, Magnetic resonance imaging

## Abstract

**Purpose:**

This study aims to evaluate the effect of dimercaptosuccinic acid (DMSA)-coated superparamagnetic iron oxide (γ-Fe_2_O_3_@DMSA) bearing the 2-deoxy-d-glucose (2-DG) ligand on targeting tumors with high-glucose metabolism.

**Procedures:**

γ-Fe_2_O_3_@DMSA and 2-DG-conjugated γ-Fe_2_O_3_@DMSA (γ-Fe_2_O_3_@DMSA-DG) were prepared. The glucose consumption of MDA-MB-231 and MCF-7 breast cancer cells and human mammary epithelial cells (HMEpiCs) was assessed. Cells were incubated with γ-Fe_2_O_3_@DMSA or γ-Fe_2_O_3_@DMSA-DG, and MDA-MB-231 cells which exhibited the highest glucose consumption were used in breast cancer xenografts. Tumor targeting was studied by magnetic resonance imaging and Prussian blue staining *in vivo*.

**Results:**

Glucose consumption was highest in MDA-MB-231 and lowest in HMEpiCs. *In vitro*, there was significant uptake of γ-Fe_2_O_3_@DMSA-DG by MDA-MB-231 and MCF-7 cells within 2 h and this was inhibited by glucose. Uptake of γ-Fe_2_O_3_@DMSA-DG was significantly higher in MDA-MB-231 compared with MCF-7 cells, and there was no obvious uptake of γ-Fe_2_O_3_@DMSA in either cell line. *In vivo*, γ-Fe_2_O_3_@DMSA-DG could be detected in the liver and in tumors post-injection, while γ-Fe_2_O_3_@DMSA was nearly undetectable in tumors.

**Conclusions:**

2-DG-coated γ-Fe_2_O_3_@DMSA improved tumor targeting of γ-Fe_2_O_3_@DMSA which can be assessed by magnetic resonance imaging.

## Introduction

In the 1920s, Warburg discovered that even in the presence of ample oxygen, cancer cells prefer to metabolize glucose by glycolysis. This is seemingly a paradox, since glycolysis, when compared with oxidative phosphorylation, is a less-efficient pathway for producing ATP [1]. This phenomenon, known as the Warburg effect, is defined as the state in which cancer cells maintain upregulated glycolysis to adapt to hypoxic conditions caused by solid aggressive tumors that overgrow the blood supply of the feeding neovasculatures *in vivo* [2]. Glycolytic metabolism requires significant amounts of glucose. Breast cancer cells increase glucose uptake by increasing the expression of the glucose transporters (GLUT), predominantly GLUT1, which transport glucose to the intracellular medium [3, 4]. This propensity is the basis behind the clinical imaging and staging of tumors via positron emission tomography (PET) [5, 6]. 2-Deoxy-2-[^18^F]fluoro-d-glucose ([^18^F]FDG) is a glucose analog that is used in PET as a diagnostic compound that targets is taken up by glucose transporters on tumors and retained in tumors by phosphorylation. This glucose analog, which permits the quantitative analysis of glycolysis, is by far the most widely used radiolabeling marker in the diagnostic work-up of primary tumors, locoregional recurrence, and distant metastases [7]. However, since [^18^F]FDG emits small amounts of radiation and CT scanners use X-rays to scan, with radiation being potentially harmful to humans, we evaluated the effectiveness of using a magnetic resonance imaging (MRI) contrast agent for tumor metabolic imaging.

MRI is a highly desirable modality for molecular imaging because it not only provides high spatial resolution but also affords excellent soft-tissue contrast. Gadolinium and superparamagnetic iron oxide (SPIO) particle-based MRI contrast agents are usually applied in clinical MRI; the former shortens the T1 time of the tissue, and the latter reduces the T2 time, thereby enhancing MRI contrast. Thus, glucose analog-modified gadolinium contrast agents have been used in MRI studies for targeting tumors [8, 9], although gadolinium-based contrast agents may cause nephrogenic systemic fibrosis and their magnetic susceptibility is low. However, SPIO particles can decrease the T2 and T2* relaxation times to achieve negative tissue contrast. Canet *et al.* demonstrated that SPIO particles also caused a strong positive contrast enhancement using T1-weighted TurboFLASH [10], thereby broadening the utility of this method. Because SPIO particles have not been shown to cause major toxicity to humans, glucose analog-labeled SPIO contrast agents have been employed for targeting tumors [11, 12].

In a recently published study using 2-deoxy-d-glucose (2-DG)-modified SPIO particles (2-DG-SPIO) coated with dimercaptosuccinic acid (DMSA) for targeting MDA-MB-231 cells, the authors found that cells treated with 2-DG-SPIO absorbed significantly more iron particles than those treated with SPIO alone [11]. In this study, we have evaluated the ability of DMSA-coated γ-Fe_2_O_3_@DMSA bearing 2-DG ligands to improve tumor detection *in vivo*.

## Materials and Methods

### Materials

Meso-2,3-DMSA was purchased from Shanghai Beihe Chemicals Co., Ltd. (Shanghai, China); 2-amino-2-deoxy-d-glucose hydrochloride (ADG·HCl) was purchased from Alfa Aesar GmbH & Co. KG (Karlsruhe, Germany); l-ethyl-3-(3-dimethylaminopropyl)carbodiimide (EDC) and *N*-hydroxysuccinimide (NHS) were purchased from Pierce Chemical Co. (Chongqing, China).

### Synthesis and Characterization of γ-Fe_2_O_3_@DMSA-DG Nanoparticles

γ-Fe_2_O_3_@DMSA-DG nanoparticles (NPs) were prepared in three steps: synthesis of γ-Fe_2_O_3_, surface coating with DMSA, and conjugation of 2-DG to the nanoparticles. γ-Fe_2_O_3_ was synthesized by chemical co-precipitation and subsequently stabilized with DMSA as previously described [12]. Briefly, γ-Fe_2_O_3_ (100 mg) was dissolved in chloroform (10 ml) and triethylamine (50 μl) was added. DMSA (50 mg) was dissolved in dimethyl sulfoxide (10 ml), which was then added to the γ-Fe_2_O_3_ solution. The resulting final solution was vortexed at 60 °C for 12 h. The resultant sample (γ-Fe_2_O_3_@DMSA NPs) constituted a black precipitate. Next, EDC (1 ml of a 0.5 mM stock) and 1 ml NHS (2.5 mM) were added to 10 ml of γ-Fe_2_O_3_@DMSA NPs (1 mM), followed by the addition of distilled water (8 ml). Following incubation for 30 min at room temperature, d-glucosamine HCl (10 ml) was added, and after 2 h, the solution was purified by overnight dialysis against distilled water (dialysis tubing, MW = 8000–10,000). The final sample was γ-Fe_2_O_3_@DMSA-DG NPs. Details of the synthesis can be obtained in a report from our research group [13].

### Glucose Consumption Assay

The glucose metabolic rate of MDA-MB-231 and MCF-7 breast cancer cells and human mammary epithelial cells (HMEpiCs) was assessed using the glucose oxidase-peroxidase (GLU-GOD) assay. Cells were seeded at a density of 5 × 10^5^ cells/well in 6-well plates and cultured for 24 h, after which the culture medium was diluted 10-fold with phosphate-buffered saline (PBS). As a control, primary culture medium for the respective cell lines was also diluted 10-fold and measured in parallel. Each data point was the average of six samples. The glucose metabolic rate was calculated using the formula [(*G*_0_–*G*_24_) × *l*] / *N*, where *G*_0_ is the glucose concentration of the primary culture medium, *G*_24_ is the glucose concentration of the medium after cells were cultured for 24 h, *l* is the quantity of culture medium, and *N* is the number of seeded cells.

### In Vitro Analysis of 2-DG-SPIO Uptake in Breast Cancer and HMEpiCs

MDA-MB-231 and MCF-7 cells were purchased from the cell bank of the Chinese Academy of Sciences (Shanghai, China), and HMEpiCs and culture medium (MED-0001, SUP-0001) were purchased from PriCells (Wuhan, China). MDA-MB-231 and MCF-7 cells were cultured in DMEM supplemented with 10 % fetal bovine serum in a humidified atmosphere of 5 % CO_2_ at 37 °C. HMEpiCs were cultured in MED-0001 supplemented with SUP-0001. Cells were incubated with 5 ml of culture medium containing γ-Fe_2_O_3_@DMSA-DG NPs or γ-Fe_2_O_3_@DMSA NPs at an iron concentration of 0.3 μmol/ml for different time intervals (10 min, 30 min, 1 h, and 2 h). Competition experiments were performed by adding 25 mmol/l d-glucose to γ-Fe_2_O_3_@DMSA-DG NPs non-glucose DMEM or MED-0001 growth medium. After incubation, the culture medium was removed. Adherent cells were washed three times with PBS (0.1 mol/l, pH 7.4), trypsinized, and centrifuged for 5 min at 2000×*g*. The number of cells was determined using a Neubauer counting chamber. For Prussian blue staining, cells were seeded in six-well plates on glass coverslips. A total of 1 × 10^6^ cells were embedded in gelatin (500 μl, 0.3 % *v*/*v* final) at room temperature for MRI measurement; 150 μl of the cell suspension was transferred into a 96-well plate, and the absorbance was read at 480 nm using a microplate reader (model 680; Bio-Rad, OK, USA).

### Prussian Blue Staining

Cell monolayers grown on glass coverslips were washed three times with PBS and subsequently fixed with methanol and acetone (−20 °C). The fixed cells were incubated with 10 % potassium ferrocyanide for 5 min, incubated with 10 % potassium ferrocyanide in 20 % hydrochloric acid for 30 min, and counterstained with nuclear fast red.

### Determination of Intracellular Iron Content

An ultraviolet colorimetric assay was performed as previously described [14]. Briefly, cells were washed with PBS, trypsinized, and dissolved in 30 % *v*/*v* HCl at 60 °C for 2 h. Any ferrous chloride present was oxidized to ferric chloride using 0.1 mg/ml ammonium persulfate (10 ml). Then, the K_4_[Fe(CN)_6_]·3H_2_O solution (20 ml) was added for 10 min to form the iron-thiocyanate complex. An aliquot of this mixture (150 μl) was transferred into a 96-well plate, and the absorbance was read at 480 nm using a microplate reader. A standard curve was generated using FeCl_3_·6H_2_O solutions of different concentrations under the same conditions. Each experiment was repeated five times.

### MRI and T2 Relaxometry of Cells in Gelatin

T2 MR relaxometry of cells in gelatin was performed using a multi-echo spin echo pulse sequence [time of repetition (TR) = 3000 ms; time to echo (TE) range = 22, 44, 66, 88, …, 330, and 352 ms, 16 echoes; field of view (FOV) = 150 × 73 mm; matrix = 256 × 256; slice thickness = 3 mm; voxel size = 9 mm^3^]. T2 relaxometry was tested using a T2 map. The T2 map calculation can be found under “dynamic analysis,” a mono-exponential fit without signal offset. The detailed formula is as follows:

$$ {T}_2\left(x,y\right)=-\frac{{\displaystyle \sum {w}_i{\displaystyle \sum T{E_i}^2{w}_i-{\left({\displaystyle \sum T{E}_i{w}_i}\right)}^2}}}{{\displaystyle \sum \ln}\left(I{\left(x,y\right)}_{A,}\right){w}_iT{E}_{i\kern0.5em }{\displaystyle \sum {w}_i-{\displaystyle \sum T{E}_i{w}_i}}{\displaystyle \sum \ln \left(l{\left(x,y\right)}_A\right)}{w}_i} $$

where TE_*i*_ is the echo time of image *I* and *w*_*i*_ is the weight of image *i* with ${w}_i=\frac{1}{{\mathrm{TE}}_i}$.

### MTT Cytotoxicity Test

Cell viability was measured using the 3-(4,5-dimethylthiazol-2-yl)-2,5-diphenyltetrazolium bromide (MTT) assay. MDA-MB-231 cells (approximately 10^5^ cells/well) were cultured in 96-well plates for 2 days. The culture medium was then removed, 100 μl of growth medium containing γ-Fe_2_O_3_@DMSA-DG NPs or γ-Fe_2_O_3_@DMSA NPs (0.3–2.4 μmol/l Fe) was added to each well, and the cells were incubated for 24 h. For each concentration group, 10 wells were measured and 10 wells were left untreated as controls. Following incubation, 10 μl (5 mg/ml) of MTT solution was added to each well and the plate was incubated for 2 h. The absorbance of each well was measured at 570 nm on an ELISA reader. Cell viability was expressed as a percentage of control ([*A*]test / [*A*]control × 100 %).

### Targeting Human MDA-MB-231 Breast Cancer Xenografts

MDA-MB-231 cells were selected for xenograft experiments as these cells consume more glucose than MCF-7 cells. All experiments were approved by the governmental review committee on animal care. Human MDA-MB-231 breast cancer xenografts were generated by subcutaneous injection of MDA-MB-231 cells (2 × 10^5^ cells/mouse) in the right neck dorsal region of female nude (BALB/c) mice (*n* = 24) (Vital River, Beijing, China). Animals were injected with a contrast agent formulation for imaging when the tumors were approximately 1 cm in diameter, which occurred approximately 4 weeks after injection of tumor cells. Animals were anesthetized by intraperitoneal injection of diazepam (8 μg/g) (Jiangsu Jumpcan Pharmaceutical Group Co., Ltd., Jiangsu, China) and ketamine hydrochloride (120 μg/g) (Jiangsu Hengrui Medicine Co., Ltd., Jiangsu, China).

### Contrast Agent Administration

Mice with MDA-MB-231 tumors (*n* = 6) were slowly injected with γ-Fe_2_O_3_@DMSA-DG NPs (300 μmol Fe/kg) in 1000 μl sterilisata (two tail vein injections of 500 μl with a 30-min interval between injections). MRI was performed at six intervals (before injection, 1 h, 6 h, 12 h, 24 h, and 48 h post-injection). Control animals (*n* = 6) were injected with γ-Fe_2_O_3_@DMSA NPs at the same dose of Fe, using the same method.

### MRI and Analysis

MRI experiments were performed using a 1.5-T whole-body MR scanner (MAGNETOM Avanto 1.5 T; Siemens, Munich, Germany) within a wrist coil. MRI was performed in a coronal plane with a T2W turbo-spin-echo sequence (TR = 5500 ms; TE = 100 ms; FOV = 150 × 73 mm; matrix = 256; slice thickness = 3 mm; flip angle = 170°; selected B1 filter, prescan normalized for the coil and image filter).

### Prussian Blue Staining

Mice (*n* = 12) were anesthetized and euthanized by cervical dislocation 24 h after injection. Frozen sections of the tumor, brain, liver, and muscle were fixed in paraformaldehyde (−20 °C). For Prussian blue staining, the fixed tissues were incubated in 10 % potassium ferrocyanide for 5 min and then incubated in 10 % potassium ferrocyanide in 20 % hydrochloric acid for 30 min, followed by counterstaining with nuclear fast red.

### Statistical Analysis

All numeric data are expressed as mean ± standard error of the mean. A standard unpaired *t* test was performed. Analysis of variance, followed by *a posteriori* Fisher test, was used to test for differences in T2 signal intensity (or T2 value) of the brain, liver, muscle, and tumor after contrast medium injection within the different groups of mice. *p* values less than 0.05 were considered statistically significant. All statistical analyses were performed with SPSS software, version 17.0 (SPSS Inc., Chicago, USA).

## Results

### Nanoparticle Characterization

The dispersion of γ-Fe_2_O_3_@DMSA NPs and γ-Fe_2_O_3_@DMSA-DG NPs in water produced nanoparticle aggregates. We previously reported [12] that the average hydrodynamic diameter of γ-Fe_2_O_3_@DMSA NPs and γ-Fe_2_O_3_@DMSA-DG NPs was 154.6 ± 28.3 and 156.2 ± 28.2 nm, respectively, which was the total diameter of aggregates and their aqueous layer thickness. Our earlier work [12] also suggests that no larger aggregates were generated during the conjugation reaction.

The nanoparticle characterization was reported [12] as follows: most particles were quasi-spherical; the average diameter of a single NP core was 10 nm; the γ-Fe_2_O_3_@DMSA-DG NPs were γ crystal; DG was successfully functionalized onto the surface of γ-Fe_2_O_3_@DMSA NPs; the molar ratio between Fe and DMSA was 100:2.5; the numerical ratios between Fe_2_O_3_ NPs and DMSA and DMSA and ADG were 1:516 and 1:1.2, respectively; the percentage of 2-DG on the surface of γ-Fe_2_O_3_@DMSA NPs was approximately 60 % (carboxyl group: 2-DG = 10:6); and the saturation magnetization values (*M*_s_) for γ-Fe_2_O_3_@DMSA NPs and γ-Fe_2_O_3_@DMSA-DG NPs were 48.95 and 49.67 emu/g, respectively.

### γ-Fe_2_O_3_@DMSA NPs and γ-Fe_2_O_3_@DMSA-DG NPs Uptake in Breast Cancer Cells and HMEpiCs

The level of glucose consumption by MDA-MB-231 cells ([1.39 ± 0.19] × 10^−4^ μmol/cell) was higher than that of MCF-7 cells ([1.12 ± 0.21] × 10^−4^ μmol/cell) after 24 h, whereas HMEpiCs exhibited the lowest levels of glucose consumption ([7.13 ± 0.08] × 10^−5^ μmol/cell). The difference in glucose consumption was significant between the three cell lines (*p* < 0.01).

**Fig. 1:**
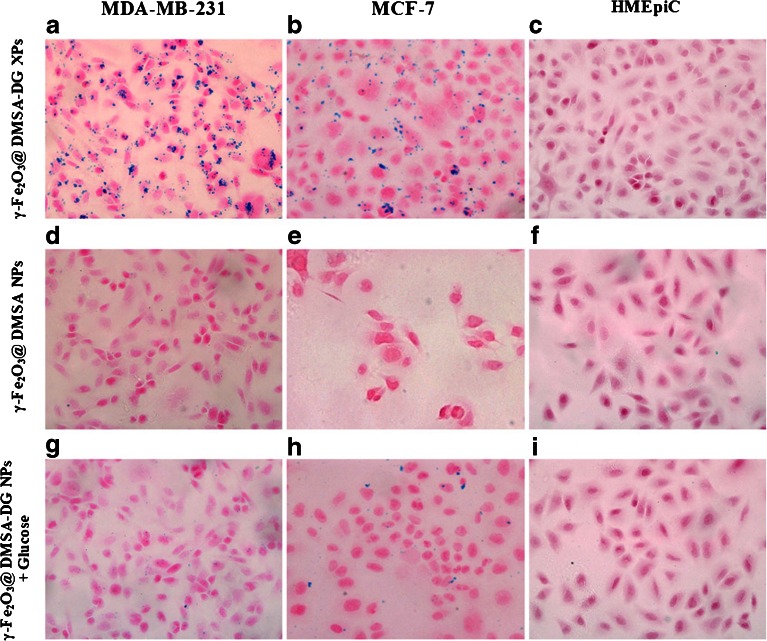
Prussian blue staining in MDA-MB-231 incubated with *a* γ-Fe_2_O_3_@DMSA-DG NPs, *d* γ-Fe_2_O_3_@DMSA NPs, and *g* γ-Fe_2_O_3_@DMSA-DG NPs plus glucose for 30 min; MCF-7 incubated with *b* γ-Fe_2_O_3_@DMSA-DG NPs, *e* γ-Fe_2_O_3_@DMSA NPs, and *h* γ-Fe_2_O_3_@DMSA-DG NPs plus glucose for 30 min; and HMEpiCs incubated with *c* γ-Fe_2_O_3_@DMSA-DG NPs, *f* γ-Fe_2_O_3_@DMSA NPs, and *i* γ-Fe_2_O_3_@DMSA-DG NPs plus glucose for 30 min. *a* MDA-MB-231 cells exhibited significantly more uptake than *b* MCF-7 cells, and there were no blue granules in the cytoplasm of *c* HMEpiCs. *d* MDA-MB-231 cells, *e* MCF-7 cells, and *f* HMEpiC incubated with γ-Fe_2_O_3_@DMSA NPs displayed almost no granules in the cytoplasm. Glucose transporters were blocked by addition of free glucose, followed by incubation with γ-Fe_2_O_3_@DMSA-DG NPs. Blue granules in the cytoplasm of *g* MDA-MB-231 and *h* MCF-7 cells decreased significantly compared with *a* and *b*, respectively.

The uptake of γ-Fe_2_O_3_@DMSA NPs, γ-Fe_2_O_3_@DMSA-DG NPs, and γ-Fe_2_O_3_@DMSA-DG NPs after blocking the target by addition of d-glucose was assessed by Prussian blue staining in cells. After 10 min, strong uptake of γ-Fe_2_O_3_@DMSA-DG NPs was observed in MDA-MB-231 cells and MCF-7 cells. Analysis of blue granules in the cytoplasm of MDA-MB-231 cells revealed stronger uptake of γ-Fe_2_O_3_@DMSA-DG NPs compared with MCF-7 cells. Blocking the glucose receptor with free glucose effectively reduced the absorption of γ-Fe_2_O_3_@DMSA-DG NPs in the cytoplasm of MDA-MB-231 cells and MCF-7 cells, indicating that the accumulation of these particles was specifically mediated by binding of glucose to the glucose transporter (Fig. 1). There was no significant uptake of γ-Fe_2_O_3_@DMSA NPs, γ-Fe_2_O_3_@DMSA-DG NPs, or γ-Fe_2_O_3_@DMSA-DG NPs in the presence of glucose in HMEpiCs in less than 2 h, whereas slight uptake of γ-Fe_2_O_3_@DMSA NPs was observed in MDA-MB-231 and MCF-7 cells after 1 h.

**Fig. 2:**
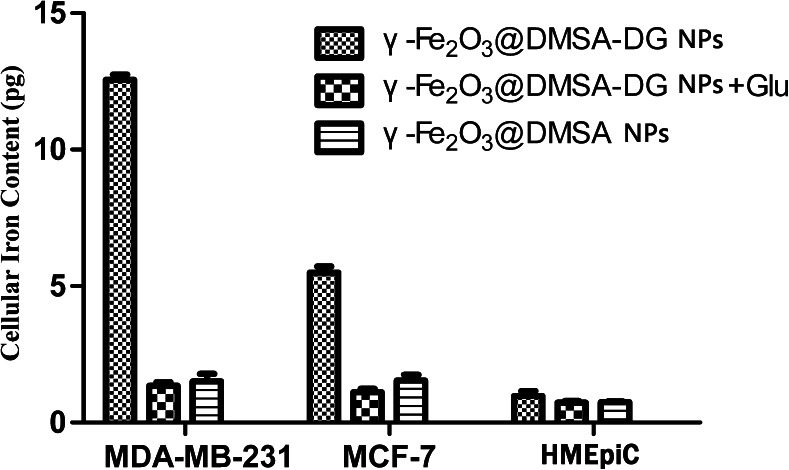
Cellular iron content in MDA-MB-231, MCF-7, and HMEpiCs incubated with γ-Fe_2_O_3_@DMSA-DG NPs, γ-Fe_2_O_3_@DMSA NPs, and γ-Fe_2_O_3_@DMSA-DG NPs plus glucose for 30 min, as determined by an ultraviolet colorimetric assay.

Quantification of cellular iron content by ultraviolet colorimetric assay demonstrated a higher uptake of γ-Fe_2_O_3_@DMSA-DG NPs compared with γ-Fe_2_O_3_@DMSA NPs and γ-Fe_2_O_3_@DMSA-DG NPs after blocking the glucose receptor by glucose incubation for the same length of time, in MDA-MB-231 cells and MCF-7 cells (*p* < 0.05). However, there were no significant differences between the cellular iron content of γ-Fe_2_O_3_@DMSA NPs and γ-Fe_2_O_3_@DMSA-DG NPs in HMEpiCs (*p* > 0.05). The ultraviolet colorimetric assay also revealed higher uptake of γ-Fe_2_O_3_@DMSA-DG NPs in MDA-MB-231 cells compared with MCF-7 cells, while HMEpiCs had the lowest uptake value after the same incubation time (Fig. 2).

**Fig. 3:**
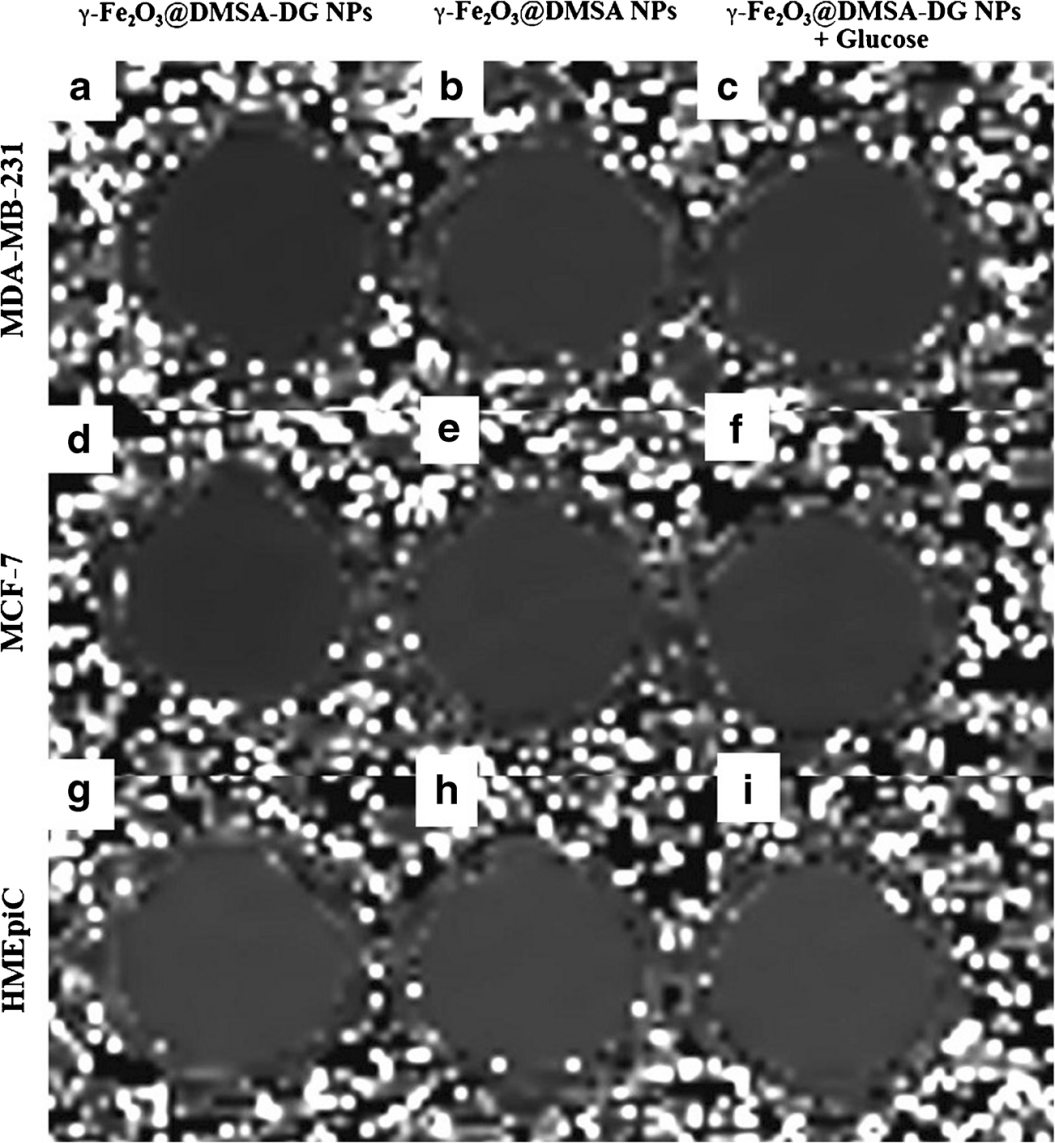
Relaxation time mapping of MDA-MB-231, MCF-7, and HMEpiCs incubated with γ-Fe_2_O_3_@DMSA-DG NPs, γ-Fe_2_O_3_@DMSA NPs, and γ-Fe_2_O_3_@DMSA-DG NPs plus glucose for 30 min. The samples of *a* MDA-MB-231 and *d* MCF-7 incubated with γ-Fe_2_O_3_@DMSA-DG NPs are shown in *black*, indicating significant iron particle absorption. Other samples (*b*, *c*, *e*–*i*) are displayed in *gray*, indicating no significant iron particle absorption.

These changes were also reflected in the T2 relaxation times. After 10 min of incubation, the mean T2 relaxation time of MDA-MB-231 and MCF-7 cells exposed to γ-Fe_2_O_3_@DMSA-DG NPs was significantly shorter than that of cells incubated with γ-Fe_2_O_3_@DMSA NPs (*p* < 0.05), while there were no significant difference in the mean T2 relaxation time change in the different groups of HMEpiCs (*p* > 0.05). After blocking the binding site with free glucose and subsequent incubation with γ-Fe_2_O_3_@DMSA-DG NPs, the T2 relaxation times of MDA-MB-231 and MCF-7 cells were significantly higher than those of MDA-MB-231 and MCF-7 cells incubated with γ-Fe_2_O_3_@DMSA-DG NPs in non-glucose medium (Fig. 3, Table 1). Furthermore, the T2 relaxation time of MDA-MB-231 cells was significantly shorter than that of MCF-7 cells incubated with γ-Fe_2_O_3_@DMSA-DG NPs after the same length of incubation (*p* < 0.05).

**Table 1:** T2 relaxation times of MDA-MB-231, MCF-7, and HMEpiCs incubated with γ-Fe_2_O_3_-DMSA-DG NPs, γ-Fe_2_O_3_-DMSA NPs, and γ-Fe_2_O_3_-DMSA-DG NPs + glucose (mean ± SD)

Cells	Incubation time (min)	γ-Fe_2_O_3_@DMSA-DG NPs	γ-Fe_2_O_3_@DMSA NPs	γ-Fe_2_O_3_@DMSA-DG NPs + Glu
MDA-MB-231	10	248.40 ± 7.74	294.56 ± 4.42	292.60 ± 3.69
	30	202.12 ± 7.22	272.88 ± 11.12	272.78 ± 7.84
	60	69.26 ± 6.50	205.20 ± 29.01	224.62 ± 24.60
	120	58.66 ± 12.71	164.60 ± 22.30	151.06 ± 15.74
MCF-7	10	260.82 ± 9.25	292.26 ± 9.13	292.76 ± 10.27
	30	219.90 ± 8.92	289.88 ± 4.42	287.86 ± 6.36
	60	172.96 ± 11.72	278.66 ± 7.98	270.68 ± 11.34
	120	175.52 ± 14.45	266.08 ± 5.59	266.78 ± 4.55
HMEpiC	10	299.20 ± 9.55	298.16 ± 6.96	297.02 ± 9.28
	30	298.20 ± 6.42	294.98 ± 7.25	296.98 ± 6.90
	60	288.80 ± 6.81	288.88 ± 6.28	288.94 ± 8.54
	120	282.96 ± 8.23	284.36 ± 7.13	279.88 ± 10.17

The viability values of MDA-MB-231 cells treated with γ-Fe_2_O_3_@DMSA-DG NPs were 100.23 ± 2.26 (0.3 μmol/ml), 100.19 ± 2.43 (0.6 μmol/ml), 100.11 ± 1.69 (1.2 μmol/ml), 99.48 ± 2.54 (2.4 μmol/ml), and 99.75 ± 2.73 (4.8 μmol/ml). The viability of cells treated with γ-Fe_2_O_3_@DMSA NPs was 99.64 ± 1.73 (0.3 μmol/ml), 99.76 ± 2.72 (0.6 μmol/ml), 99.61 ± 1.98 (1.2 μmol/ml), 99.18 ± 2.21 (2.4 μmol/ml), and 97.31 ± 4.02 (4.8 μmol/ml). One-way analysis of variance revealed no significant differences in cell viability between the two groups of treated cells (*p* > 0.05). The MTT assay did not demonstrate reduced viability of MDA-MB-231 cells after incubation with γ-Fe_2_O_3_@DMSA NPs or γ-Fe_2_O_3_@DMSA-DG NPs, at variable iron concentrations for 24 h. These results indicate that γ-Fe_2_O_3_@DMSA NPs and γ-Fe_2_O_3_@DMSA-DG NPs do not inhibit cell growth at Fe concentrations between 0.3 and 2.4 μmol/ml and therefore caused no significant cytotoxicity.

### Detection of Targeted Tumors

#### 1.5-T Dynamic MRI

**Fig. 4:**
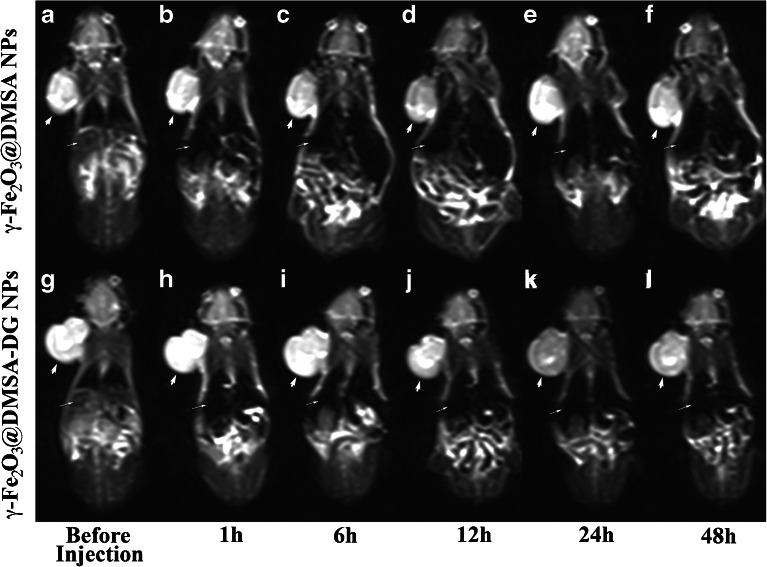
1.5-T MRI turbo-spin-echo-T2-weighted (5500/100) dynamic imaging of human MDA-MB-231 breast cancer xenografts. *a*–*f* Injection of γ-Fe_2_O_3_@DMSA NPs showed that the tumor (*thick white arrows*) signal intensity decreased at 12 h which returned to basal levels by 24 h. *g*–*l* Injection of γ-Fe_2_O_3_@DMSA-DG NPs showed that the tumor signal intensity decreased between 12 and 48 h, with the most hypointensity observed at 24 h. The signal intensity in the liver (*thin white arrows*) significantly decreased after injection of γ-Fe_2_O_3_@DMSA NPs or γ-Fe_2_O_3_@DMSA-DG NPs.

**Fig. 5:**
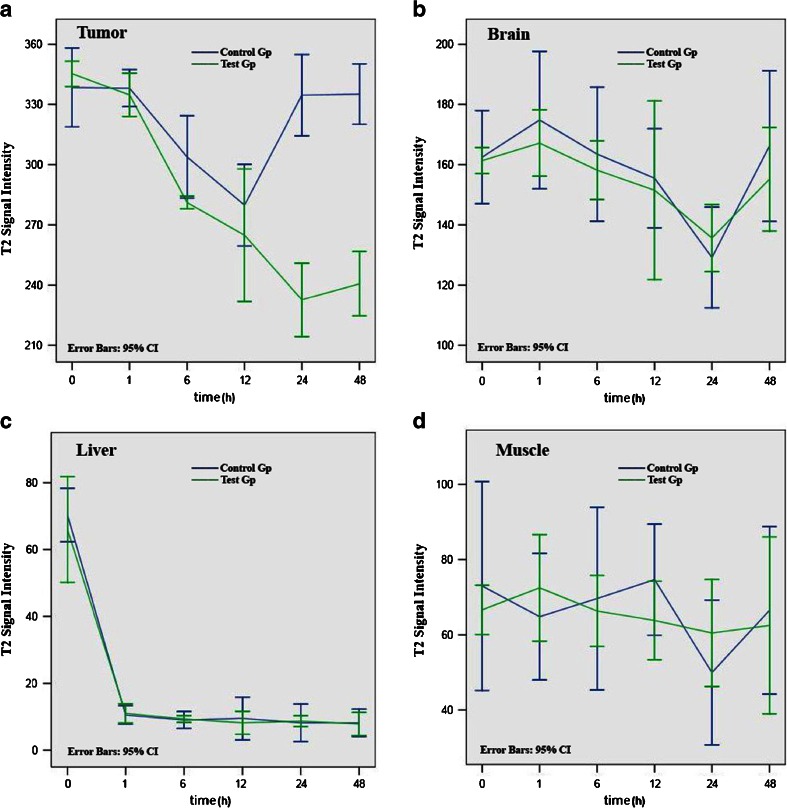
T2 signal intensity in the **a** tumor, **b** brain, **c** liver, and **d** muscle (mean ± standard error of the mean) after injection of γ-Fe_2_O_3_@DMSA NPs (*blue line* control group) and γ-Fe_2_O_3_@DMSA-DG NPs (*green line* test group). In the tumor, the predominant T2 signal intensity decreased at 24 and 48 h in the γ-Fe_2_O_3_@DMSA-DG NPs group, which was significantly different to the γ-Fe_2_O_3_@DMSA NPs group. There were no significant differences between the two groups with regard to the brain, liver, and muscle.

Tumors exhibited different MRI features in the two different study groups. In the γ-Fe_2_O_3_@DMSA NPs group, MRI T2-weighted images showed a decrease in tumor signal intensity 12 h after injection, which returned to basal levels before injection after 24 h (Fig. 4). In the γ-Fe_2_O_3_@DMSA-DG NPs group, the T2 weighted images exhibited a decrease in tumor signal intensity 12–48 h after injection, with the most significant hypointensity occurring 24 h after injection (Fig. 4). The signal intensity in the liver significantly decreased 1 h after injection of γ-Fe_2_O_3_@DMSA NPs or γ-Fe_2_O_3_@DMSA-DG NPs, and after which point, the liver retained its hypointensity. Signal intensity decreased slightly in the brain at 24 h; however, the mean signal intensity of each time point in the two groups was not significantly different in the brain and the signal intensity in the muscle was not significantly different pre-injection compared with 48 h post-injection (Fig. 5).

#### Uptake of γ-Fe_2_O_3_@DMSA NPs and γ-Fe_2_O_3_@DMSA-DG NPs in Tissues

**Fig. 6:**
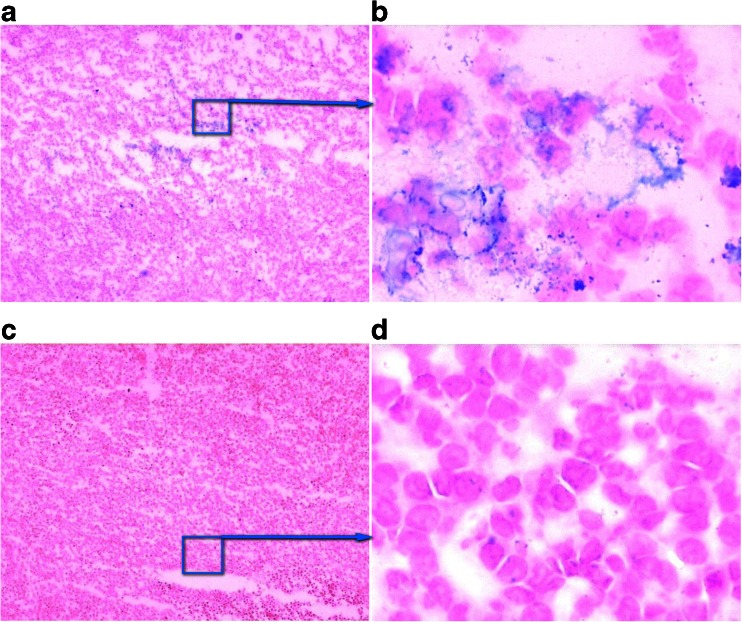
Frozen tumor tissue sections with Prussian blue staining 24 h after injection of γ-Fe_2_O_3_@DMSA-DG NPs (**a**, **b**) and γ-Fe2O3@DMSA NPs (**c**, **d**). The was uptake of iron particles (*blue*) in tumor cells surrounding the necrotic tumor tissue (**a** × 1; **b** × 40), but there was no uptake of iron particles in tumor tissue after injection of γ-Fe_2_O_3_@DMSA NPs (**c** ×1; **d** ×40).

Twenty-four hours post-injection, Prussian blue staining of tumor tissues in the γ-Fe_2_O_3_@DMSA NPs group (*n* = 6) revealed no significant uptake of γ-Fe_2_O_3_@DMSA NPs in tumor cells (Fig. 6). In contrast, iron particle uptake was observed in tumor cells around the necrotic site in the γ-Fe_2_O_3_@DMSA-DG NPs group (*n* = 6) (Fig. 6). An abundance of iron particles was also observed in the liver, but no iron particles were found in the muscle and brain.

## Discussion

Previous studies in which iron oxide particles accumulate in the reticuloendothelial system but not in tumor cells have been challenged. Indeed, SPIO particles penetrating the capillary endothelium and tumoral uptake have been observed [15, 16], particularly when used at higher doses. These observations are also supported *in vitro*, in cell culture studies [17].

In this study, DMSA-coated and 2-DG-conjugated SPIOs were developed and their ability to target breast cancer cells with high-glucose consumption was investigated both *in vitro* and *in vivo*. Our results suggest that this design allows significantly higher *in vivo* tumor enhancement compared with unconjugated 2-DG iron oxide nanoparticles in a human breast cancer xenograft model.

The pharmacokinetics and cellular uptake of SPIOs *in vivo*, including their ability to manage biological barriers, are largely related to NP physicochemical properties, including morphology, hydrodynamic size, charge, and other surface properties [18, 19]. NPs consist of a magnetically active core coated with a stabilizing shell to which target ligands or ligand analogs, and additional imaging modalities are anchored, which makes them preferable for biological applications. DMSA coating provides a significant advantage in that the presence of carboxyl groups on the particle surface can easily be used to covalently attach specific ligands or ligand analogs, and the coating layer, which does not contribute to the MRI signal, can be kept very thin.

The exploitation of GLUT overexpression in tumor cells and tissues, such as the glucose receptor GLUT1, has already been successfully harnessed in PET studies with fluorodeoxyglucose or [99mTc]-DTPA-DG [20–22] and has been used in MRI with Gd-DTPA-DG [9] and niosomes bearing glucose conjugates (*N*-palmitoyl glucosamine) [8]. Glucose receptors are overexpressed in many tumor cells because of their enhanced metabolism [3, 23]. In this study, we assessed glucose consumption in MDA-MB-231 and MCF-7 breast cancer cells and HMEpiCs for 24 h using the GLU-GOD assay. We found that MDA-MB-231 cells exhibited the highest level of glucose consumption, while glucose consumption by HMEpiCs was significantly lower than that of MCF-7 cells. Data from our *in vitro* study showed that the uptake of iron particles in MDA-MB-231 cells was greater than that of MCF-7 cells following incubation with γ-Fe_2_O_3_@DMSA-DG NPs for the same period of time. There was no significant iron particle uptake in HMEpiCs incubated with γ-Fe_2_O_3_@DMSA-DG NPs within 2 h. To prove that the particles were absorbed through the glucose receptors, we blocked the glucose receptor with free glucose in MDA-MB-231 and MCF-7 cells and then incubated the cells with γ-Fe_2_O_3_@DMSA-DG NPs. This led to a significant decrease in the cellular uptake of iron particles, suggesting that the uptake of γ-Fe_2_O_3_@DMSA-DG NPs by MDA-MB-231 and MCF-7 cells occurs via glucose receptors that are overexpressed in these cells.

According to other reports, expression of GLUT1 occurs predominantly around the area of tumor necrosis [24, 25]. Our xenograft models had tumor diameters of approximately 1 cm, because such large tumors usually contain necrotic areas. Targeted NP drug delivery systems have shown faster and higher levels of accumulation in tumors compared with non-targeted systems [26–29]. In this study, following administration of γ-Fe_2_O_3_@DMSA-DG NPs in mice, 1.5-T MRI revealed that the T2 signal intensity in tumors decreased from 12 to 48 h. This was significantly different from the results observed after administration of γ-Fe_2_O_3_@DMSA NPs, where the T2 signal intensity in tumors decreased at 12 h but returned to basal levels prior to 24 h. Notably, there were no significant differences in T2 signal intensity between the two groups in the brain, liver, and muscle. Prussian blue staining of tumor tissues revealed iron particle uptake in some tumor cells 24 h after injection of γ-Fe_2_O_3_@DMSA-DG NPs, whereas no significant iron particle uptake was observed in tumor cells 24 h after injection of γ-Fe_2_O_3_@DMSA NPs. This demonstrates that the targeting NPs prepared in this study had tumor-specific targeting aggregation. Thus, the addition of a targeting molecule onto the surface of NPs increased the selective cellular binding and internalization through receptor-mediated active uptake and endocytosis. Without the incorporation of targeting ligands or ligand analogs, NPs only rely on nonspecific interactions with cell membranes.

The results of *in vitro* experiments may differ significantly to those obtained *in vivo. In vitro*, a number of particles may surround the tumor cells; however, *in vivo*, after intravenous injection, numerous opsonin proteins, cells, and salts bind to the surface of the nanoparticles. This is known as the opsonization process and is responsible for easier recognition by the mononuclear phagocytic system leading to rapid clearance from the circulation. Nanoparticles are usually taken up by macrophages in the liver [Kupffer cells (80–90 %), spleen (5–8 %), and bone marrow (1–2 %)]. After uptake in specialized macrophages, SPIOs are degraded lysosomally. The nanoparticles internalized by tumor cells cannot be metabolized and eliminated quickly. The coating material is eliminated via other decomposition and elimination pathways. For example, the coating of ferumoxtran-10 is degraded via intracellular dextranases and is primarily eliminated renally (89 % in 8 weeks) and the core material is supplied to the iron storage pool of the body.

The γ-Fe_2_O_3_@DMSA-DG NPs or γ-Fe_2_O_3_@DMSA NPs used were non-monodispersed SPIOs, as they were not connected to active surface molecular polyethylene glycol and, as such, could not avoid phagocytosis by the reticuloendothelial system (RES) in the body. They do not have identical physical properties for uncontrolled bio-distribution and bio-elimination. The average hydrodynamic diameter of the nanoparticle was approximately 150 nm, and a large number of these particles were taken up by the RES. In our *in vivo* study, we observed that the T2-weighted signal in the liver decreased immediately after injection of γ-Fe_2_O_3_@DMSA-DG NPs or γ-Fe_2_O_3_@DMSA NPs, indicating that most particles were phagocytized by the RES. If the 2-DG-modified SPIO decreases the both signals of liver metastasis and the health parts, the liver tumor may not be easily detected.

However, the biocompatibility of such particles is not only related to their nano-size but is particularly related to their surface properties, which are the determining factors for cellular uptake and cytotoxicity [26]. In addition, coating materials also play an important role [27], as coating serves to further functionalization of the particles by ligands or ligand analogs, helping to facilitate receptor-mediated endocytosis or phagocytosis [28, 29].

## Conclusion

In this study, DMSA-coated naked iron oxide supplied carboxyl groups, which were linked with d-glucosamine hydrochloride and X-ray spectroscopy demonstrated that DG was successfully functionalized onto the surface of γ-Fe_2_O_3_-DMSA NPs. The glucose analog, 2-DG, can bind to glucose transporters overexpressed in tumor cells. Our results demonstrated the uptake of γ-Fe_2_O_3_@DMSA-DG NPs by breast cancer cells exhibiting high-glucose consumption, but no uptake of γ-Fe_2_O_3_-DMSA NPs. These findings indicate that it is possible to non-invasively identify high-glucose metabolic tumors by MRI after injection of γ-Fe_2_O_3_@DMSA-DG NPs. However, compared with ^18^F-FDG PET-CT, one drawback of this approach is that it requires two MRI scans (before injection and 24 h after injection).
